# Treatment with a Zinc Metalloprotease Purified from *Bothrops moojeni* Snake Venom (BmooMP-Alpha-I) Reduces the Inflammation in an Experimental Model of Dextran Sulfate Sodium-Induced Colitis

**DOI:** 10.1155/2019/5195134

**Published:** 2019-07-29

**Authors:** Maraisa Cristina Silva, Helioswilton Sales-Campos, Carlo José Freire Oliveira, Tamires Lopes Silva, Flávia Batista Ferreira França, Fábio Oliveira, Tiago Wilson Patriarca Mineo, José Roberto Mineo

**Affiliations:** ^1^Laboratório de Imunoparasitologia Dr. Mário Endsfeldz Camargo, Instituto de Ciências Biomédicas, Universidade Federal de Uberlândia, 38400-902 Uberlândia, MG, Brazil; ^2^Laboratório de Imunologia, Instituto de Ciências Biológicas e Naturais, Universidade Federal do Triângulo Mineiro, 38025-180 Uberaba, MG, Brazil; ^3^Departamento de Biociências e Tecnologia, Instituto de Patologia Tropical e Saúde Pública, Universidade Federal de Goiás, 74605-050 Goiânia, GO, Brazil; ^4^Laboratório de Biofísica, Instituto de Ciências Biomédicas, Universidade Federal de Uberlândia, 38400-902 Uberlândia, MG, Brazil; ^5^Instituto Nacional de Ciência e Tecnologia em Nano-Biofarmacêutica (N-Biofar), 31270-901 Belo Horizonte, MG, Brazil

## Abstract

It has been described that the metalloprotease BmooMP-alpha-I purified from *Bothrops moojeni* snake venom is able to hydrolyze the TNF molecule. However, this observation has been based mainly on *in vitro* investigation, in addition to molecular modeling and docking approaches. Considering that there is no in vivo study to demonstrate the biological effects of this enzyme, the major aim to the present work was to investigate whether the BmooMP-alpha-I has any anti-inflammatory efficacy by setting up a murine experimental design of colitis induced by dextran sulfate sodium (DSS). For this purpose, C57BL/6 mice were divided into six groups, as follows: (i) animals without intestinal inflammation, (ii) animals without intestinal inflammation treated with BmooMP-alpha-I (50 *μ*g/animal/day), and (iii) animals with intestinal inflammation induced by 3% of DSS, (iv) mice with intestinal inflammation induced by DSS and treated with BmooMP-alpha-I enzyme at the 50, 25, or 12.5 *μ*g/animal/day dosages by intraperitoneal route. Clinical signs of colitis were observed daily for calculating the morbidity scores, cytokine measurements, and histological features. We observed that the animals treated with different doses of the enzyme presented a remarkable improvement of colitis signs, as confirmed by a significant increase of the intestine length in comparison to the DSS group. Also, no difference was observed between the groups treated with the enzyme or vehicle, as the colon length of these animals was slightly lower than that of the group of healthy animals, without induction of intestinal inflammation. The cytokine quantification in supernatants of intestinal tissue homogenates showed a significant reduction of 38% in IFN-gamma levels, when the animals were treated with 50 *μ*g of the BmooMP-alpha-I compared to the animals receiving DSS only. A significant reduction of 39% in TNF levels was also observed in all doses of treatment with BmooMP-alpha-I, in addition to a significant reduction of 35% in the amount of IL-12p40. Histological examinations revealed that the BmooMP-alpha-I 50 *μ*g treated group preserved colon architecture and goblet cells and reduced the ulcer area, when compared with DSS mice, which showed typical inflammatory changes in tissue architecture, such as ulceration, crypt dilation, loss of tissue architecture, and goblet cell depletion, accompanied by a significant cell infiltration. In conclusion, our results suggest that the improvement of clinical scores and histological findings related to BmooMP-alpha-I treatment in this experimental model could be attributed to the metalloprotease ability to modulate cytokine production locally at the inflamed intestine. These findings highlight the potential anti-inflammatory role and effectiveness of this enzyme as a therapeutic alternative in this type of immunopathological condition.

## 1. Introduction

Inflammatory bowel diseases (IBD), such as ulcerative colitis (UC) and Crohn's disease (CD), are chronic inflammatory disorders identified by an imbalance between inflammatory and regulatory immune responses at the gut mucosa [1]. The incidence of inflammatory bowel diseases has increased worldwide, and it is now estimated that between 1 and 1.3 million Americans are currently diagnosed with IBD [2–4]. This increased incidence is possibly due to currently unidentified environmental factors, which interact with an inherent genetic predisposition and immune dysregulation [4–6]. Ulcerative colitis (UC) is an intestinal inflammation that presents extensive damage of colon mucosa. The various clinical manifestations with attacks of abdominal cramps, pain, bloody diarrhea, rectal bleeding, weight loss, fever, and easy fatigability may begin gradually or start totally all at once [5–7]. These clinical manifestations interfere notably in the quality of life of affected patients [8].

Cytokines have a crucial role in the pathogenesis of IBD, because these immunological markers are able to guide multiple aspects of the inflammatory response and they are associated with the clinical symptoms. The combination of genetic and environmental characteristics is necessary to initiate alterations in epithelial barrier function, thereby leading to the translocation of luminal pathogens or even components from commensal microbiota into the bowel wall. Subsequently, unbalanced cytokine responses in such microenvironmental niche trigger subclinical or acute mucosal inflammation mostly in genetically susceptible hosts. In patients that fail to control the acute intestinal inflammation, chronic intestinal inflammation develops, which is the result of an uncontrolled activation of the mucosal immune system. Furthermore, cytokines seem to have a crucial role in the pathogenesis of progressive and destructive forms of IBD, being associated with complications such as intestinal stenosis, rectal bleeding, abscess and fistula formation, and the development of colitis-associated neoplasia [9].

Pharmacological and/or biological treatment strategies for IBD are used in the clinical management of the patients, which include aminosalicylates, corticosteroids, apremilast, cytokine inhibitors (such as IL-6–IL-6R or IL-12–IL-23), modulators of cytokine signaling events (such as JAK inhibitors or SMAD7 blocker), inhibitors of transcription factors (such as GATA3 or ROR*γ*t), anti-T-cell activation and antimigration factors (such as *β*7 integrin), or TNF blockers (anti-TNF antibodies) [10, 11]. However, all these strategies are not totally effective and a significant number of patients require repeated intestinal surgeries to control the disease complications. Moreover, some of them are refractory to the pharmacological interventions, which may also induce severe side effects [12, 13]. In view of this, the investigations of new therapies that combine efficacy, effective dosing, and fewer adverse effects are an important goal for human IBD therapy. In this regard, the use of alternative therapies has emerged as a helpful approach to treat this type of gastrointestinal diseases, because it has been described that almost half of the IBD patients have attempted or presently use alternative therapies [13, 14]. In fact, there are different complementary therapies, such as the use of bioactive factors, particularly those derived from plants and animals, that can modulate the microenvironment of intestinal inflammation [15–17], and the experimental colitis models have been used to identify this type of therapeutic agents and elucidate the underlying physiologic mechanisms of IBD. In this context, the major aim of the present study was to examine the effect of a zinc metalloprotease derived from *Bothrops moojeni* snake venom (BmooMP-alpha-I) as a potential therapeutic agent to treat intestinal inflammation in a murine model of colitis induced by dextran sulfate sodium, considering that this enzyme can interfere in the TNF biological function by directly promoting its hydrolysis.

## 2. Materials and Methods

### 2.1. Animals

C57BL/6 male mice (18-22 g/6–8 weeks old) were housed at the Rede de Biotérios de Roedores from the Universidade Federal de Uberlândia (REBIR-UFU) in temperature-controlled rooms, receiving water and food *ad libitum* throughout the experimental conditions. This study was approved by the Ethical Committee for Animal Experimentation from Universidade Federal do Triângulo Mineiro (CEUA-UFTM) (protocol # 372/16, from April 29, 2016), in accordance with the procedures established by the Universities Federation for Animal Welfare (UFAW).

### 2.2. Purification and Biochemical Characterization of BmooMP-Alpha-I

The metalloprotease BmooMP-alpha-I was isolated and purified and its biological activity was assessed, as previously described [18]. The purified enzyme was diluted, dialyzed against 50 mM ammonium bicarbonate (pH 7.8), lyophilized, and stored at -20°C until used. Protein concentration was determined by the Bradford method [19].

### 2.3. Experimental and Control Groups in DSS-Induced Colitis

**Figure 1:**
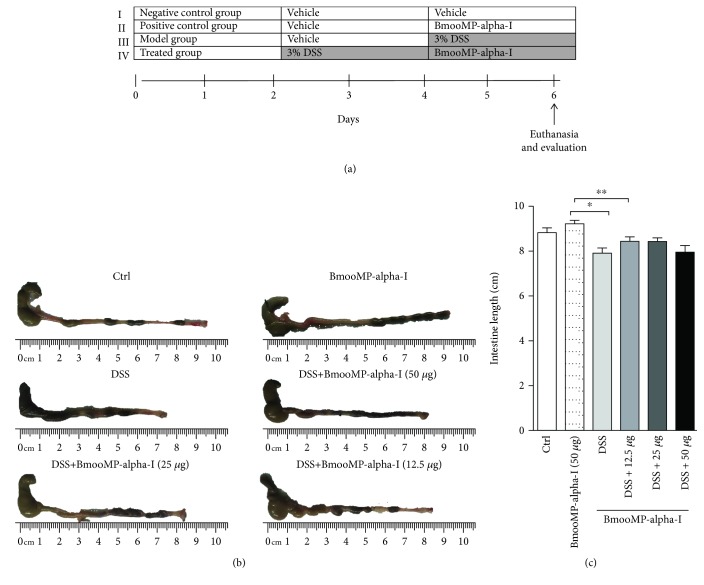
Macroscopic features at the colon. (a) The murine experimental design was set up to induce colitis by DSS administration. For the experiments, mice were divided into groups of six animals each, receiving the following inocula: I: sterile saline (vehicle, negative control); II: 50 *μ*g of BmooMP-alpha-I (positive control); III: DSS only (model group); IV: DSS plus BmooMP-alpha-I, at the doses of 50, 25, or 12.5 *μ*g/animal/day (treated group). At the end of 6 days, mice from all groups were subject the clinical evaluation and euthanized. (b) Control group (without DSS), BmooMP-alpha-I (50 *μ*g) group, 3% DSS administration group (DSS), and 3% DSS plus BmooMP-alpha-I (50 *μ*g to 12.5 *μ*g). (c) Intestine length (cm). Data shown are expressed as mean ± SD (*n* = 6 per group). Results represent means ± SD out of five analyses for each experimental condition. Comparisons were carried out by using one-way ANOVA and Dunnett's Multiple Comparison Test. ^∗^*p* < 0.05 and ^∗∗^*p* < 0.01; ns: not significant in relation to the DSS or negative control (Crtl).

Experimental design for DCC-induced colitis was carried out in accordance with our previous work [20]. Mice were allocated into six groups with six animals each, as follows: animals without intestinal inflammation (negative control group); animals without intestinal inflammation treated with BmooMP-alpha-I (50 *μ*g/animal/day) (treatment control group); animals with intestinal inflammation induced by 3% DSS (M.W. 36,000-50,000, MP Biomedicals, Illkirch, France), treated with the diluent only (PBS) (vehicle group); and mice with intestinal inflammation induced by DSS and treated with the BmooMP-alpha-I enzyme at the doses of 50, 25, or 12.5 *μ*g/animal/day (treatment groups). Each mouse from all groups was identified on its tail by a nontoxic pen. Mice from the model control group were exposed to 3% (*w*/*v*) DSS in drinking water during 6 consecutive days. After being exposed to DSS for 6 days, mice from the treatment groups were treated intraperitoneally (i.p.) with BmooMP-alpha-I and euthanized at day 6 after the beginning of the DSS exposure. Animals from the control groups were treated with vehicle only during the 6 days (Figure 1(a)).

### 2.4. Clinical Signs and Score Determinations

Clinical signs of colitis were determined, as previously described by our group [20]. Briefly, the following parameters were evaluated: weight loss, wet anus, diarrhea, bleeding stools, hypoactivity, and piloerection. The disease scores were evaluated according to the same criteria defined in our previous work [20]. Mice were euthanized at day 6 after colitis induction, and the intestines were collected for further analysis. Intestinal tissues were immediately frozen in liquid nitrogen in the presence of a protease inhibitor cocktail (Complete®, Roche Pharmaceuticals, Mannheim, Germany) for myeloperoxidase (MPO) activity.

### 2.5. Cytokine Measurements

After euthanasia, the colon was portioned into 4 pieces weighting 60 mg/each on average. To facilitate comparison of data between mice and groups, the same portions were used for each determination. TNF was quantified using a commercial ELISA kit (R&D Systems, Minneapolis, MN, USA). Also, the levels of IL-12p40 and IFN-gamma production were determined in these samples, using appropriate kits (BD, Franklin Lakes, NJ, USA). The cytokine concentrations in the intestinal samples were determined in accordance with the manufacturer's instructions.

### 2.6. MPO Assay

Fragments from the colon were cut into small pieces, weighed, and processed for MPO quantification, as previously described [21], with modifications. Briefly, for the MPO assay, fragments were homogenized and erythrocytes were lysed. The pellets obtained after centrifugation were resuspended, followed by three freezing and thawing rounds. After centrifugation, the supernatants were transferred to 96-well plates and the MPO measurements were carried out by addition of a TMB Substrate Reagent Set (tetramethylbenzidine, BD OptEIA™, San Diego, CA) at 37°C. The reaction was stopped, and readings were performed in a spectrophotometer at 450 nm. The results were expressed as optical density per gram of tissue.

### 2.7. Histology and Histopathological Analysis

To observe the effect of the metalloprotease treatment in the DSS-induced colitis, the intestinal tissues from all groups of animals were obtained and analyzed, as previously described [20]. Briefly, the final portions of the colon were opened longitudinally for assessment of macroscopic damage. These tissues were rinsed in PBS, placed in 10% buffered formalin for 24 h, and processed to be embedded in paraffin. Tissue sections of 5 *μ*m were acquired and processed for hematoxylin and eosin staining. Images were obtained at 20x magnification with an FSX100 Olympus Microscope.

### 2.8. Statistical Analysis

Data were analyzed by using GraphPad Prism software (La Jolla, CA, USA). As the results showed normal distribution, the one-way ANOVA test followed by the Bonferroni post hoc test was performed. The results are representative from at least three independent experiments, and the statistical analysis was based on 6 animals per group. Values of *p* < 0.05 were considered significant.

## 3. Results

**Figure 2:**
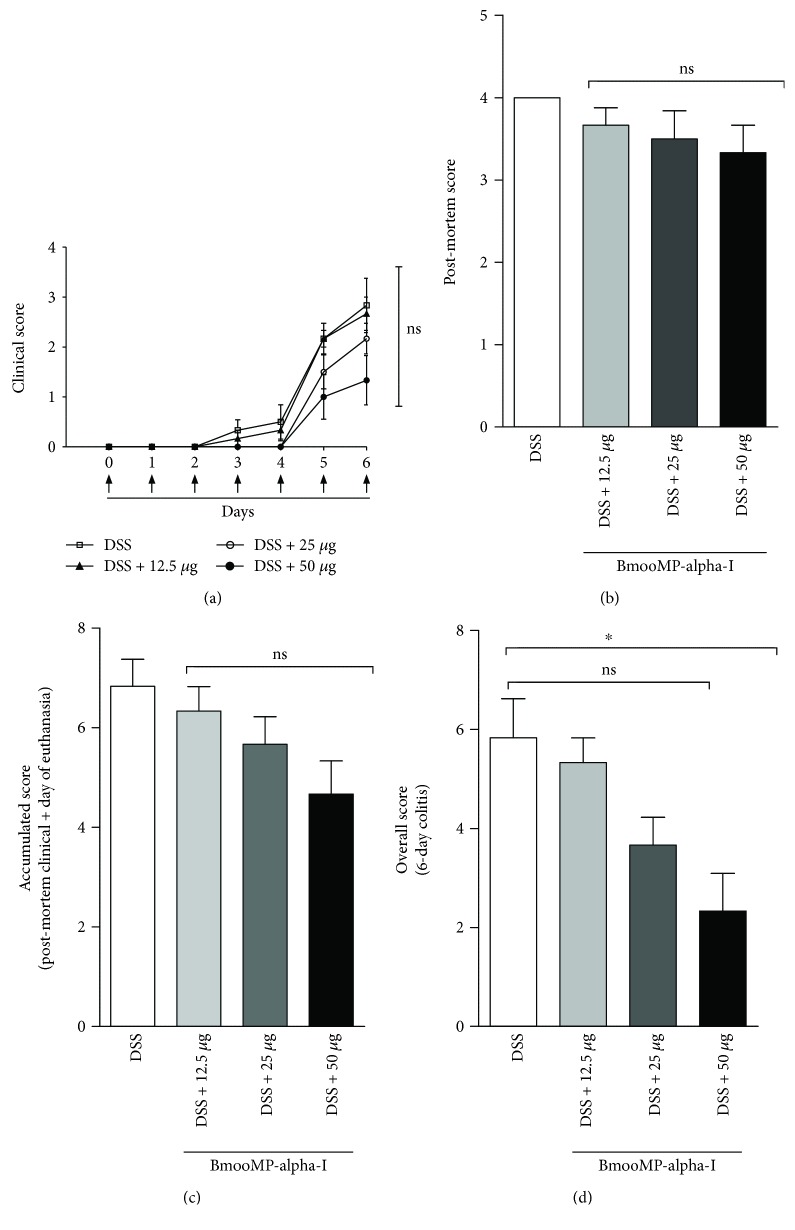
Effect of BmooMP-alpha-I on the clinical signs of mice in DSS-induced colitis model: (a) clinical score; (b) post-mortem score; (c) accumulated score; (d) overall score. Results represent means ± SD out of five analyses for each experimental condition. Comparisons were carried out by using one-way ANOVA and Dunnett's multiple comparison test. ^∗^*p* < 0.05. ns: not significant in comparison with the DSS group of animals.

To evaluate whether the BmooMP-alpha-I enzyme would be able to interfere in the colitis inflammatory signs, C57BL/6 mice were treated with 50, 25, or 12.5 *μ*g/animal/day by intraperitoneal route of the enzyme and the disease clinical outcome was evaluated as described in the design experiment (Figure 1(a)). Even though significant difference was not observed under macroscopic evaluation of the intestine at the time of euthanasia (Figure 1(b)), the clinical signs showed marked improvements, as the animals treated with the highest enzyme dose had an increased colon length, which was similar to that observed in the vehicle-treated group or in the other doses tested. Also, no difference was observed between the groups treated with the enzyme or vehicle, as the colon length of these animals was slightly lower than that of the group of healthy animals, without induction of intestinal inflammation (Figure 1(c)). Additionally, we showed that mice treated the highest dose of BmooMP-alpha-I showed a tendency to reduce the clinical score, post-mortem score, and accumulated score when compared to the other groups (Figure 2(a)–2(c)). The beneficial effect the highest dosage of BmooMP-alpha-I was confirmed by the overall score, which represents the clinical condition of the mice during all periods of the experimental observation, considering that significant differences were observed among the treated groups and the colitis group (Figure 2(d)).

**Figure 3:**
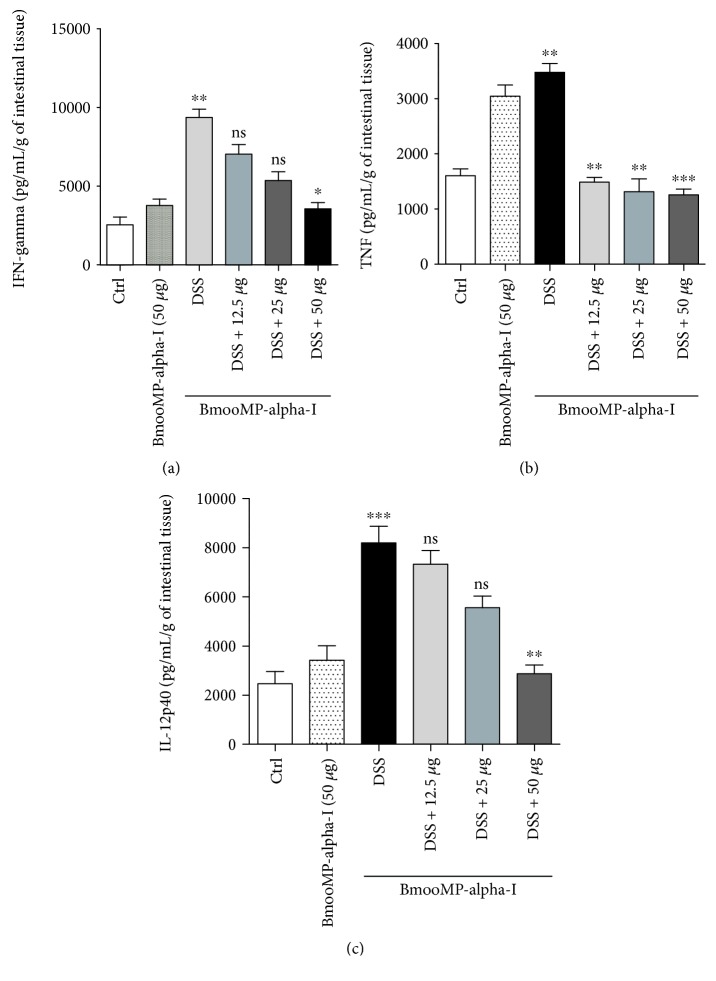
Effect of BmooMP-alpha-I on cytokine production in DSS-induced colitis: (a) TNF, (b) IFN-gamma, and (c) IL-12p40 concentration in supernatants of intestinal tissue homogenates. Results represent means ± SD out of five analyses for each experimental condition. Comparisons were carried out by using one-way ANOVA and Dunnett's multiple comparison test. ^∗^*p* < 0.05, ^∗∗^*p* < 0.01, and ^∗∗∗^*p* < 0.001. ns: not significant when compared with the DSS or negative control (Ctrl) groups.

To assess the effect of BmooMP-alpha-I in the production of proinflammatory cytokines and myeloperoxidase (MPO) activity, additional experiments were performed. The cytokine measurements in the supernatants of intestinal tissue homogenates showed a significant reduction of 38.0% in IFN-gamma levels, when the animals were treated with 50 *μ*g/animal/day of the BmooMP-alpha-I compared to the animals receiving DSS only (Figure 3(a)). Also, significant reductions of the TNF levels in the animals treated with BmooMP-alpha-I in all doses tested were observed, with a reduction average of 38.9% compared to that of the DSS-inducted colitis group (Figure 3(b)). Furthermore, the highest dose of BmooMP-alpha-I was also able to significantly reduce by 35.0% the amount of IL-12p40 (Figure 3(c)).

On the other hand, the measurements of MPO levels demonstrated that the animals with colitis and treated BmooMP-alpha-I were not statistically significant compared to the untreated DSS-induced colitis group. Overall, these results suggested that the improvement of clinical scores related to the BmooMP-alpha-I therapy could be attributed to its ability to modulate cytokine production locally at the inflamed intestine.

**Figure 4:**
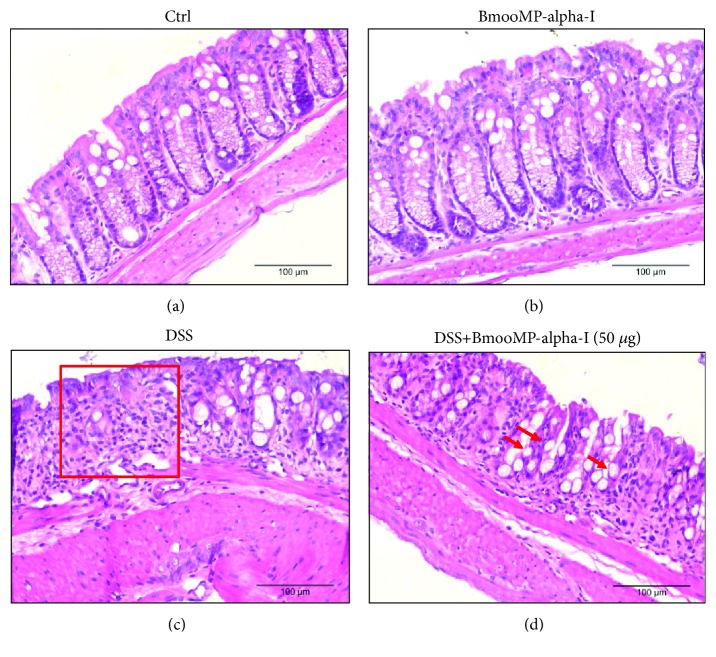
Histological characteristics of the colon from mice obtained at day 6 after induction of the DSS colitis model in comparison with those of the controls. H&E staining was performed on colon sections obtained from mice belonging to the following groups: (a) negative control group (no DSS), (b) positive control group (50 *μ*g BmooMP-alpha-I), (c) model group (3% DSS), and (d) treated group (3% DSS + 50 *μ*g BmooMP-alpha-I). Images were obtained at 10x magnification with an FSX100 Olympus Microscope. The square demonstrates the loss of tissue architecture and cell infiltration. The arrows indicate the goblet cells.

Histological analyses of the colon revealed that DSS mice showed typical inflammatory changes in colon morphology, characterized by crypt dilation, goblet cell depletion, and inflammatory cell infiltration, resulting in a significant loss of tissue architecture. Compared to the DSS group, mice treated with the highest dose of BmooMP-alpha-I presented an ameliorated histological aspect of the tissue and reduced inflammatory signs. The enzyme did not cause tissue damage, presenting the same aspect of the negative control group (Figure 4(a)–4(d)).

## 4. Discussion and Conclusions

The inflammatory disorders affecting the intestinal tissues, as ulcerative colitis and Crohn's disease, remain as severe gastrointestinal diseases to be solved [22, 23]. Currently, therapeutic advances have led to a paradigm shift in the clinical management of patients with IBD. The introduction of immunosuppressive or biologic agents, such as azathioprine or TNF blockers, has markedly reduced the need to use corticosteroids for therapy [24–26]. However, one of the most investigated approaches that has been described is based on the treatment with antibodies directed to TNF. Currently, monoclonal antibodies against TNF are commercially available to treat TNF-mediated pathologies. In fact, these antibodies are also frequently applied for treatment of rheumatoid arthritis, in addition to promising results that have been obtained for the treatment of other inflammatory disorders [22, 23]. Concerning the inflammatory diseases affecting the intestinal tissues, however, the anti-TNF antibody therapy has been described to be moderately effective during clinical management of the patients. Indeed, there are several observations pointing out that not all patients respond to the anti-TNF therapy, besides the fact that this therapeutic option has been associated with serious side effects [27]. TNF is a classical proinflammatory cytokine involved in the modulation of acute inflammatory responses and host defense mechanisms [27]. It is mainly produced by monocytes and secreted as a transmembrane protein (mTNF-26 kDa) and cleaved by the TNF-converting enzyme (TACE), a zinc metalloprotease, in its soluble form (sTNF-17 kDa). Both fragments are biologically active and bind as trimers to both TNFR1 and TNFR2 receptors [28]. However, it is necessary to point out that TNF has pleiotropic effects in the bowel wall: it induces neoangiogenesis, activates macrophages to produce proinflammatory cytokines, favors Paneth cell death via necroptosis, augments apoptosis of intestinal epithelial cells, regulates T-cell apoptosis, and reduces production of the tissue inhibitor of matrix metalloproteinases (MMPs) by fibroblasts to mediate tissue injury via activated MMPs. Thus, anti-TNF antibodies can suppress intestinal inflammation in IBD through several mechanisms [9, 29].

In our previous study, we investigated the role of BmooMP-alpha-I as a biological product able to hydrolyze the TNF cytokine, based mainly on in vitro experiments, as well as on modeling and docking approaches [18]. We concluded that this metalloprotease could be used to modulate of the inflammatory response, but this application would require further investigation, particularly when tested to treat inflammatory disorders involving the TNF cytokine. Consequently, in the present study, we advanced in the process to characterize this metalloprotease as a modulator of the inflammatory response, by using an in vivo experimental design already applied in our group to assess an experimental inflammatory bowel disease [20]. Therefore, when compared with our previous studies [18, 20], it is necessary to emphasize that the present work investigated for the first time the use of the BmooMP-alpha-I metalloprotease to treat DSS-induced colitis.

The efficacy of the BmooMP-alpha-I was assessed by setting up a murine experimental design of colitis induced by DSS. The clinical signs of colitis were observed daily for calculating the disease scores, and it was found that the animals treated with different doses of this enzyme presented a marked improvement of colitis signs, as observed by an increase in the intestine length. Also, no difference was observed between the groups treated with the enzyme or vehicle, as the colon length of these animals was slightly lower than that of the group of healthy animals, without induction of intestinal inflammation. The association between exposure to DSS and decrease in the bowel length constitutes an important piece of information [26, 30], and this parameter has been recently reinforced in the literature, demonstrating the colon length as a well-established indicator of DSS-induced colitis, particularly when the colon shortening is examined after 7 days of DSS treatment. In the present work, as predicted, DSS treatment resulted in colon shortening, whereas the group of animals treated with BmooMP-alpha-I significantly improved such an indicator of inflammatory response.

The cytokine quantification in supernatants of the intestinal tissue homogenates showed a significant reduction of 39% in TNF levels when the treatment with BmooMP-alpha-I was performed. A significant reduction of 38% in IFN-gamma levels was also observed, when the animals were treated with 50 *μ*g of the BmooMP-alpha-I compared to the animals receiving DSS only. In addition, a significant reduction of 35% in the amount of IL-12 was also observed in our DSS-induced colitis, even though it has been described that the effect of the BmooMP-alpha-I metalloprotease on TNF is independent of cell cytotoxicity and it does not affect other TLR-triggered cytokines, such as IL-12p40 [25].

Histological examinations revealed that the group treated with 50 *μ*g of BmooMP-alpha-I preserved colon architecture and goblet cells and reduced the ulcer area, when compared with DSS mice, which showed typical inflammatory changes in the tissue morphology, such as crypt dilation, loss of tissue architecture, and goblet cell depletion, accompanied by significant cell infiltration.

Metalloproteases represent at least 30% of the toxin composition of many viperid snake venoms, and they are responsible for hemorrhage through disturbances in the blood coagulation cascade of prey and snakebite victims [31]. However, certain metalloproteases lack hemorrhagic activity but present other biological effects, such as inhibition of platelet aggregation, induction of apoptosis, and pro- or anti-inflammatory activities [32, 33]. A previous study of the crystal structure of BmooMP-alpha-I showed that the enzyme presents a catalytic zinc ion displaying an unusual octahedral coordination, which includes three canonical histidines [34]. In the present work, we clearly demonstrated that the treatment with BmooMP-alpha-I metalloprotease could impair the inflammatory response in the DSS-induced colitis experimental design. In summary, our results suggest that the improvement of clinical scores and histological findings related to BmooMP-alpha-I treatment in this experimental model could be attributed to the metalloprotease ability to modulate cytokine production locally at the inflammatory intestinal microenvironment. We conclude that these findings could provide a novel perspective to treat intestinal inflammatory diseases, highlighting the potential anti-inflammatory role of this metalloprotease and its effectiveness as a therapeutic alternative in this type of immunopathological condition.

## Data Availability

The data used to support the findings of this study are available from the corresponding author upon request.
